# Boundary debates: The new challenge of Psychiatry

**DOI:** 10.4103/0019-5545.64577

**Published:** 2010

**Authors:** Philip John

**Affiliations:** Peejays Child Guidance Clinic, Cochin, S. India and CGC, Sharjah, UAE

## CHANGING BOUNDARIES

“*The Boundary between Behavior and Biology is arbitrary and changing. It has not been imposed by the natural contours of Disciplines, but by lack of knowledge at the time*”

(E.R. Kandel)

## BODY-MIND DUALISM

The boundary, the dichotomy between body and mind held sway for a long time. This Cartesian dualism achieved a shrewd truce between the church in the 17^th^ century and science, by partitioning body and mind into two domains. Such a dichotomy then appeared logical – what was ‘visible’ went to science and what then was ‘invisible’ went to the church and the charlatans. This shrewd and clever truce enabled spectacular progress in all other disciplines of medicine except the ‘science of mind’, which simply could not have progressed asking more of philosophical and sociological questions than biological.[1]

Even as the science of mind subsequently progressed, its boundaries have been under perpetual violation and incursion – by empiricisms in the past decades, and ‘overlapping’ fraternity at present because we cannot, as yet, clearly define this business of ‘mind’.

With the exponential technology available today that creates windows to ‘peep’ into live brain cells as they go about performing the mental functions, the mind may appear as arising purely from physical processes such as electrical signals or complex cellular chemicals. On a different plane, the mind transcends the physical, as something evanescent and ethereal that may be closer to the philosophical or spiritual concept of the soul! So, here we are – does the soul belong in psychiatry, neuro-theology, philosophy or theology? The boundaries are still indistinct as indubitably expressed by Prof. Mike Trimble, Professor of behavioral neurology at the Institute of Neurology, London, in his recent book - ‘The Soul in the Brain’. However, when your temperament and training are right, “if you open up a brain, the mind will eventually fall out”.[2]

## PSYCHOLOGY AND THE ISOLATION OF PSYCHIATRY

The dualism between psychology and psychiatry too is a derivative of the faded mind-brain issue, which once created professional contest and personal vanity within the field of behavioral sciences.

Psychiatrists must never discard or diminish the environmental influences in the etiopathogenesis of psychiatric disorders. Nevertheless, those in our own profession who pretend that psychiatry is not a branch of medicine, in order to facilitate ‘multi-disciplinary approach’, contributed to the abysmal isolation of psychiatry from the rest of medicine.[3] As a consequence, even physicians sometimes fail to distinguish psychiatrist from a psychologist!

While training at National Institute of Mental Health and Neuro Sciences (NIMHANS), many in our generation went through these boundary tensions. But fortunately, those were related to ‘hierarchical’ disputes and the movement that we then spearheaded settled those issues once and for all. Even in a multi disciplinary system, the hierarchical order cannot be compromised at any cost; in fact hierarchy only strengthens a holistic approach to management.[4]

Technology has transformed the operating boundaries between psychology and psychiatry in the clinical practice of medicine, and ended psychiatry’s isolation. Mind being generated by the nervous system, we discern that psychiatry is a pure branch of medicine. Psychiatry should be perceived and practiced as application of basic neurosciences to man’s day-to-day problems.[1] Therefore, psychiatrists are primarily physicians. Period.

## BOUNDARY BETWEEN BEHAVIOR AND BIOLOGY

Expanding knowledge of the roots of behavior in neurobiology is fast merging frontiers and obliterating boundaries between related disciplines such as neurology, psychiatry and pediatrics. As Kandel implied, the arbitrary boundaries between behavior and biology are not imposed by contours of the medical disciplines. Therefore, when it comes to medical practice today, there is unabated encroachment on the clinical boundary of Psychiatry from many a related discipline.

There are practical reasons for this maneuvering into psychiatry. At least a third of a primary care physician’s prescriptions may involve a psychiatric drug. A third of all epileptics would also hold a psychiatric diagnosis. A major overlap in most medical disciplines comes from Psychiatry.

Merging of boundaries has made it possible for allied medical disciplines to give a name to most psychiatric disorders, right or wrong, and get on with treatment. The stigma that a psychiatric consultation may arouse for the patient is another reason to withhold a referral.

The emergence of the ‘new science of mind’ and its neurological substrate has finally enabled exciting breakthroughs in psychopharmacology. These advances created the availability, by design and not serendipity, of successful drugs in psychiatry which any medical specialist can freely prescribe in India even without adequate empowerment. This advent of increasingly specific and safe psychopharmacological options which anyone can freely use is another reason for the unabating encroachment on the practice of psychiatry from related disciplines.

Biological boundaries between neurology and psychiatry, or clinical boundaries between pediatrics and psychiatry are being increasingly obliterated. My objective is to catalyze, complement and harmonize trans-disciplinary relationships, not to maim or mutilate them. This emotion is central to my presentation.

## NEUROLOGY AND PSYCHIATRY: STATIC AND DYNAMIC

Firing of neurons moves a limb; firing neurons fabricate fear or fervor. Behind a crooked behavior, there is a crooked neuron! We ‘think and feel’ from inside the brain. The distinction between psychiatry and neurology may thus be artificial or absurd.

Neurologists conventionally focus on the ‘static’, on the demonstrable pathology. Psychiatrists may focus on the ‘dynamic’, on the ‘function-al’.[3] All mental activities are created by parallel, dynamic, biological events in the brain, through a process of central coherence.

Neurology and psychiatry come together in the human brain, the most complex object in the whole universe. These many billions of cells mysteriously regulate the body (wherein the neurologists transact), learn and direct our behavior, store and interpret a life-time of experiences, summon the thoughts and memories unique and personal to each individual (wherein the psychiatrists are operative). Thus the neurologists’ and the psychiatrists’ common world is the brain – the essence of what makes us human, and whole.

## TYROSINE TO TERRORISM

The new science of mind is almost a complete paradigm shift. An ordinary amino acid tyrosine is used by the creator to synthesize two powerful catecholamines that seem to determine or change the destiny of a human being or that of millions of them, by normally or abnormally impacting neural networks that control emotions within the human brain, triggering fear or violence, war or terror. Or even peace and pleasure. Practice of psychiatry and neurology must converge here, to modulate brain mechanisms to alleviate suffering and to improve quality of life in this world.

## CHILD PSYCHIATRY AND BEHAVIORAL PEDIATRICS

While basic biological issues determine the blurring or merging of boundaries between neurology and psychiatry, mere clinical overlaps with psychiatry have introduced a new dimension in the practice of pediatrics. While behavioral neurology and psychiatry operate on one and the same template, pediatrics is a medical discipline with a distinct and different temperament.

Historically, child psychiatry evolved with the child guidance delivery system starting in the 1920s. In 1946, a formal American Association of Psychiatric Clinics for Children (AAPCC) was established. In 1953, the American Academy of Child Psychiatry, an organization of medical practitioners came into being. Finally in 1959, the Academy was ‘legitimized’ as a Medical subspecialty with the establishment of Board Certification under the American Board of Psychiatry and Neurology.

During those early years, trans-disciplinary interventions like play therapy, behavioral management etc. were central to treatment in the absence of a sound medical model. With the advent of diagnostic criteria as well as specific and efficacious psychopharmacological agents for child psychiatric disorders, there is jostle and scramble on this boundary of child psychiatry from pediatric neurology, developmental pediatrics and now behavioral pediatrics!

## BEHAVIORAL PEDIATRICS

This term was first used by Prof. Robert J. Haggerty of the University of Rochester in the 1970s. The editors of the Pediatric Clinics of North America have called him the ‘Hippocrates’ of Behavioral Pediatrics in their dedication.[5]

Dr. Haggerty, in the late 1970s, began this branch to “study behavioral concerns of children from a pediatrician’s perspective, not only from a child psychiatrist’s perspective”! In the preface of 1975 Pediatric Clinics of North America, before the foregoing definition, it was defined merely as the branch “which focuses on the psychological, social and learning problems of children and adolescents”.[6,7]

Amazingly, the 2003 issues of the Pediatric Clinics of North America define behavioral pediatrics as “what the clinician does to diagnose, to treat and to prevent mental illness in children and adolescents”.[5] In which case, what remains is gladly for child and adolescent psychiatry.

The branch of ‘Behavioral Pediatrics’ has now gone on to develop its own Society and Journal.[7] In the US, it has received approval from the Accreditation Council for Graduate Medical Education, and most recently from the American Board of Pediatrics for Board Certification, similar to neonatology.

Behavioral pediatrics condescends to admit that there is “some” overlap, with child psychiatry. And that “advancements in psycho pharmacology have led to the availability of many medications to treat any variety of mental disorders including depression, anxiety, disruptive disorders (e.g. Attention Deficit Hyperactivity Disorder - ADHD), eating disorders, and others”. “Today, families expect behavioral pediatricians to look after both the medical and behavioral health aspects that affect their children and teenagers… to meet the complex needs of children, adolescents and families that they serve”.[5]

## CONTENT OF CHILD PSYCHIATRY

The range of topics under ‘behavioral pediatrics’ starts from childhood and adolescent psychological development, through sexuality, abuse and sports medicine to ADHD, anxiety and depression. As regards the content of Child and Adolescent Psychiatry, we have ended up as B team!

It is important for us in psychiatry to discern that so far psychiatry has somehow held its ground. The International Classification of Diseases (ICD) till now has kept all the above disorders in psychiatry. That need not continue to be the case if we are not vigilant.

Several questions may be asked of us. If some of these disorders, for example, result from poor central nervous system (CNS) maturation, how come they are included under psychiatry and not neurology? How can developmental disorders constitute ‘mental’ disorders? So far, we have a justification that all these disorders are quintessentially neuro-psychiatric. There is a high frequency of co-morbidity of developmental disorders with Axis I psychiatric disorders, because of which other specialties did not want to mess with them. There is also the caveat from robust data that developmental disorders pre-dispose the child heavily to later Axis I disorders. However, more research in molecular biology may blend the boundaries of disciplines further. Our leadership needs to be watchful.

## SCOPE OF CHILD PSYCHIATRY

My thesis in child psychiatry at NIMHANS, guided by wisdom nonpareil of Prof. R.L. Kapur in 1980, did not give me then the conceptual clarity that I always longed for in that subspecialty. In 30 years, my sojourn has been from a hazy discipline with poor outcomes, to specific multiaxial diagnoses and to specific pharmacological remedies. Child Psychiatry is a mature and clearer subspecialty today.

The ease with which the diagnostic process is possible today has expanded the scope of child psychiatry, alluring sister disciplines in medicine. The greater tragedy, however, is that enough number of psychiatrists themselves have not been attracted to child psychiatry, the road less travelled, yet.

## TO COMPLEMENT AND TO HARMONIZE: THE COCHIN EXPERIENCE

In the background of this debate, a time has come for psychiatry and its academic leadership to determine at least its own clinical boundaries and assert them. Quintessentially, psychiatry takes root in neurology, but is neurologic practice (behavioral neurology) synonymous with psychiatric practice? Child psychiatric disorders obviously occur in children, but can the practice of child psychiatry become the domain of emerging branches like ‘behavioral pediatrics’?

Isn’t there training and a temperament that a medical man must acquire before he practices psychiatry of any kind!

Having said all this, let me assure that my fervent work and zealous network have always been trans-disciplinary – harmonizing with pediatricians, neurologists, psychologists and language pathologists. Supremely, there should never be cause for tension among medical specialties at all; their common goal is to satisfy the unmet needs of children distressed by disorders.

Nearly two decades ago, psychiatrists and pediatricians in Cochin began to think about psychiatric disorders in children, and my strength to start a CGC then came mainly from my pediatrician colleagues here. Along with Dr. Shoba Srinath and Dr. Sekhar Seshadri at NIMHANS.

Having crossed the magical figure of five thousand children evaluated on a hierarchical multidisciplinary protocol in a decade, our huge data is undergoing evaluation. These thousands of children and families have given us the strength to posit a ‘spectrum-construct’ of childhood developmental disorders on a new mode.[8] That strength comes from harmonizing professional boundaries through related disciplines of psychiatry, psychology, language pathology, pediatrics and neurology at the ground level on a bio-psycho-social template, causing least inter disciplinary tensions. This has been the Cochin experience.[9,10]

## LEADERSHIP IN PSYCHIATRY

Academic leadership of our sister disciplines seems to have been very focused and intense on their newer subspecialties. The Indian Academy of Pediatrics last year published a book on ‘Behavioral Pediatrics’ (which was specially prepared under ‘IAP Vision’). It was not a ‘cut and paste’ job.

Leadership in psychiatry, in order to answer many of these cross border concerns, needs to take calculated pro-active actions. Placing a substantial number of child psychiatrists in the community is part solution to our boundary concerns. We need to train many of them. When will our teaching and training institutions finally allocate a place for this subspecialty to empower and initiate enough psychiatrists to be child psychiatrists?

The need for empowering pediatricians or neurologists with the knowledge and skills to identify child psychiatric disorders is undoubtedly justified. But the response to our boundary concerns lies in psychiatrists being able to liaison with them. The Pediatric Clinics of North America says, ”…. it would be an unrealistic challenge for pediatricians to provide physical and psychological care in a 15 minute office-visit. ‘Referrals’ therefore, for further evaluation and treatment need to follow with the critical duty of the pediatrician in convincing the child and parent that ‘*additional help*’ is needed“.[5]

Our own syllabus in post graduate training needs a succinct shift of emphasis, so much more to basic neuro-sciences and neurology, with greater exposure to child psychiatry and to consultation-liaison work.

If our academic leadership abdicates for example, the huge responsibility of legitimizing this nascent subspeciality, our position will be over-run by fellow professionals, due to the sheer force of numbers of children who need and seek psychiatric help. Fellow medical specialists, perhaps due to lack of time in their consultation rooms, mostly refer these children to the old school of psychologists, and possible "*additional help*" may sadly end there!

For the sake of all patients who deserve psychiatric help, I trust that pro-active measures by our collective leadership for capacity-building our young post graduates and empowering related medical disciplines to liaison and harmonize with psychiatrists, will be the new challenge of psychiatry in India.
